# Status and Challenges for Vaccination against Avian H9N2 Influenza Virus in China

**DOI:** 10.3390/life12091326

**Published:** 2022-08-27

**Authors:** Jinze Dong, Yong Zhou, Juan Pu, Litao Liu

**Affiliations:** Key Laboratory for Prevention and Control of Avian Influenza and Other Major Poultry Diseases, Ministry of Agriculture and Rural Affairs, College of Veterinary Medicine, China Agricultural University, Beijing 100193, China

**Keywords:** influenza virus, H9N2 AIV, vaccine, antigenicity

## Abstract

In China, H9N2 avian influenza virus (AIV) has become widely prevalent in poultry, causing huge economic losses after secondary infection with other pathogens. Importantly, H9N2 AIV continuously infects humans, and its six internal genes frequently reassort with other influenza viruses to generate novel influenza viruses that infect humans, threatening public health. Inactivated whole-virus vaccines have been used to control H9N2 AIV in China for more than 20 years, and they can alleviate clinical symptoms after immunization, greatly reducing economic losses. However, H9N2 AIVs can still be isolated from immunized chickens and have recently become the main epidemic subtype. A more effective vaccine prevention strategy might be able to address the current situation. Herein, we analyze the current status and vaccination strategy against H9N2 AIV and summarize the progress in vaccine development to provide insight for better H9N2 prevention and control.

## 1. Introduction

Avian influenza virus (AIV) affiliates with the genus of type A influenza virus in the *Orthomyxoviridae* family, packaged with eight negative-sense and single-strand RNA segments encoding 10 core proteins and a variable number of accessory proteins. Influenza A viruses are commonly characterized by their combinations of hemagglutinin (HA) and neuraminidase (NA), giving rise to a multitude of different subtypes, such as H5N1, H7N9 or H9N2. According to their intravenous pathogenicity index (IVPI) in chickens, AIVs are classified into high pathogenicity avian influenza viruses (HPAIVs) and low pathogenicity avian influenza viruses (LPAIVs).

H9N2 AIV was first isolated from turkeys in Wisconsin, USA, in 1966 [1]. Since then, the presence of H9N2 AIV in poultry flocks has been reported in various countries worldwide [2,3,4]. The first isolate of H9N2 AIV in China was collected from chickens in Guangdong province in 1994 [5]. Compared to H5 and H7 HPAIVs, H9N2 LPAIV infection did not induce obvious clinical signs or death in chickens. However, H9N2 infections in poultry increased their susceptibility to secondary infections with other pathogens that could cause high mortality, leading to huge economic losses [6]. Moreover, a recent study showed that co-infection of H9N2 with infectious laryngotracheitis virus live-attenuated vaccine caused enhanced pathogenicity and immunosuppression, suggesting that we need to be more concerned about H9N2 infection during vaccination [7]. In China, which is regarded as an epicenter of avian influenza viruses, the H9N2 virus has been detected in multiple avian species [8], including chickens, domestic waterfowl and pigeons [9]. Notably, H9N2 AIVs in poultry populations, especially in China, have already acquired the ability to cross species barriers and can directly infect mammals, even humans, without a need for intermediate hosts. H9N2 AIV in poultry has been transmitted to pigs [10] and dogs [11], generating variants with novel antigenic and genetic characteristics. The enlarged host range of the H9N2 virus substantially increases its possibility of transmission to mammals. Importantly, human infections with H9N2 AIV have been sporadically reported worldwide [12]. In addition, the six internal genes of H9N2 constitute a relatively stable community to be transferred into other emerging reassortants, such as the human-infecting H7N9 [13,14], H10N8 [15] and clade 2.3.4.4 human-lethal H5N6 viruses [16], which significantly threaten public health.

Influenza vaccines are one of the best tools currently available to reduce the risk of influenza infection and its associated complications, which has been proved in a variety of animals, including humans and birds. Humoral immunity, mainly based on HA and NA, utilizes secretory IgA and IgM to provide protection against the establishment of initial infection, while IgG acts to neutralize newly replicating viruses [17]. HA antibody levels have been shown to correlate with protection against infection by influenza; antibodies against NA may also correlate with protection against infection as well as causing a reduced severity of illness [18,19]. Cell-mediated immunity, on the other hand, as elicited by major histocompatibility complex (MHC) class I-restricted CD8+ cytotoxic T lymphocytes (CTLs), plays a central role in controlling influenza-virus infection [20,21]. The major antigenic targets of these cross-reactive T cells are epitopes in the highly conserved internal proteins of influenza, particularly polymerase basic protein 1 (PB1), matrix protein 1 (M1) and nucleoprotein (NP) [22]. Recently, there has been increasing consensus on the importance of T cells being present locally in the airway or parenchyma of the respiratory tract to protect against influenza. The presence of mucosal immune responses in the respiratory tract or lung is particularly important, because most severe influenza symptoms are due to lung infection [23,24,25].

Controlling the prevalence of H9N2 virus, especially in poultry, will decrease the incidence of H9N2 human infection and reduce the production of new reassortants, thus minimizing the risk of influenza pandemics caused by H9N2 AIV. We note, however, that the prevalence of H9N2 in immunized flocks indicates that the effectiveness of the H9N2 vaccine is facing a great challenge. Therefore, this review focuses on analyzing the current status of H9N2 under vaccination programs in Chinese poultry, summarizing the progress of vaccine development, and looking forward to the control of H9N2 in China in the future.

## 2. Prevalence and Antigenic Drift of H9N2 AIVs under Vaccination in China

### 2.1. H9N2 AIV in Avian and Human

**Figure 1:**
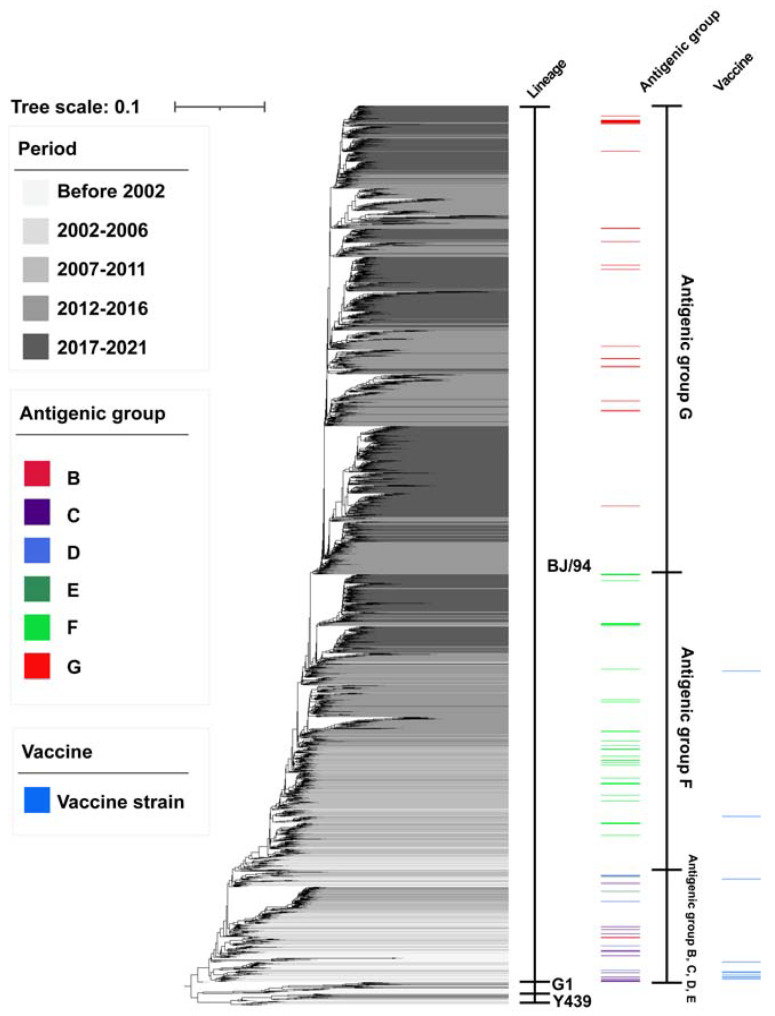
Genetic evolution of H9N2 virus in China. All available nucleotide sequences of the HA genes of H9N2 influenza virus in China as of 31 December 2021 from GISAID (www.gisaid.org) were downloaded. Duplicated sequences and sequences with 99% identity were further removed, and a total of 1923 sequences were finally gutted. Following this, the MAFFT tool was used to perform a global multiple alignment of the sequences, and the MUSCLE tool was used to perform a partial alignment to correct part of the alignment errors and manually correct some of the frameshift errors. The H9N2 virus HA gene sequences were used to construct a phylogenetic tree using IQtree software based on the maximum likelihood method. Different colors in the phylogenetic tree represent virus strains isolated during different periods; the darker the color is, the more recent the isolation time. Adobe Illustrator 2021 and Interactive Tree Of Life (iTOL) were used to annotate the strains with antigen groups and vaccines.

China, supplying over 70% of the H9N2 isolates in the database (data from GISAID), is considered the epidemic center of AIV. Since it was first reported in 1994, H9N2 AIVs have continued to circulate in China [5]. A number of reports have confirmed the widespread prevalence of H9N2 in poultry flocks in China [26,27,28,29]. Genetic evolution analysis has shown that the vast majority of H9 genes in China belong to the BJ/94-like lineage [30] (also known as the Y280-like lineage), and multiple sublineages of this lineage are currently prevalent (Figure 1). When considering all eight genes, H9N2 AIV continued to reassort and evolve to produce novel genotypes during continued circulation [31,32,33,34]. G57, a novel H9N2 genotype, was generated in 2007, triggering a widespread epidemic in poultry flocks in China, with exaggerated spread advantages [35,36,37]. The latest live-poultry-market surveillance results showed that the isolation rate of H9N2 in chickens from 2014–2019 was persistently high (~11%) and that the H9N2 subtype had gradually surpassed other subtypes to become the most predominant subtype in chicken, duck and pigeon flocks, making it the top problem in the poultry industry [38,39]. It is worth noting that such a status has occurred in the context of continuous vaccination since 1998 [33].

The widespread prevalence of H9N2 AIV in chickens increases the risk of host spillover and has public health implications. The world’s first case of human infection with H9N2 AIV was reported in Guangdong, China, in 1998 [40]. As of December 2021, global reports of laboratory-confirmed human H9N2 AIV cases had reached 95 cases, of which 32 of the 33 cases reported in 2020–2021 were from China, confirming the high risk of host spillover from the H9N2 AIV chicken epidemic [41]. Most chickens infected with H9N2 AIV do not die directly, preserving the opportunity for secondary infection with other subtypes of influenza viruses, which promotes virus reassortment. H9N2 avian influenza viruses have served as “donor viruses”, providing multiple internal genes to various subtypes of viruses, including H5N1, H5N6, H7N9, H10N8 and H10N3 [35,38,42,43,44]. Such reassortment events occurred intensively with the increased isolation rate of the G57 genotype H9N2 AIV. These novel viruses could present as regional epidemics in avian populations, or even pose a significant threat to humans [45], so controlling H9N2 AIV epidemics in avian populations is vitally important to human influenza prevention and control.

### 2.2. Antigenic Drift of H9N2 AIV under Vaccination in China

The continuous variability of H9N2 AIV antigenicity is greatly conducive to its prevalence in immunized flocks. The antigenic epitopes of HA proteins are mainly located in the head region of the trimeric protein structure, which also performs the key function of cell receptor binding [46,47]. There are four potential antigenic sites in H9-subtype hemagglutinin: Site I, Site II, H9-A, and H9-B, defined in previous studies [48,49,50,51,52]. The continued evolution of the BJ/94-like lineage of H9N2 in China has gradually produced a number of antigenic mutations, resulting in the formation of different antigenic groups. 

The first commercially available H9N2 vaccine was introduced in China in 1998, 4 years after H9N2 AIV was confirmed in chicken flocks [33]. The use of inactivated vaccines was considered to be very effective in the early stages, at least for maintaining production performance. However, most H9N2 AIVs isolated from 1997–2002 showed antigenic drift from the representative vaccine strain SD/6/96 [33]. Sun et al. classified representative H9N2 strains from 1994–2008 into five antigenic groups, groups A–E, based on their antigenic distance [30]. The distribution of antigenic groups showed an obvious correlation with the year of isolation, suggesting that H9N2 AIV was undergoing continuous antigenic changes. However, the renewal of vaccine strains is slow and lags behind the changes in antigenic groups. It usually takes approximately 5 years for a new vaccine strain to come into use. Recent findings suggest that H9N2 in China has evolved two antigenic groups with HI cross-titer differences ranging from 8- to 32-fold, with vastly different antigenicity, which can be referred to as antigenic groups F and G [37,53,54,55,56]. At present, isolates with different antigenicity are cocirculating, resulting in a mismatch between the vaccine strains and epidemic strains, which makes it difficult to effectively inhibit the spread of H9N2 AIV.

## 3. Vaccination Strategy for H9N2 AIV in China

Mass vaccination strategies, mainly using inactivated vaccines, have been implemented in China and have demonstrated satisfactory effects in controlling HPAIV [57]. H5 outbreaks have declined significantly since a mass vaccination program was initiated in 2005. HPAIV of H5 clade 7.2, which was widely circulating in northern China from 2006 to 2013 [58], has been largely eliminated by mass vaccination. In addition, mass vaccination against the zoonotic H7N9 subtype was implemented in 2017, and the isolation rate of H7N9 AIV in poultry decreased significantly. Coincidently, human cases of the sixth wave declined by 99.6% compared with the fifth wave [57]. The control strategy of H9 AIV was different from that of the H5 and H7 HPAIVs. Compulsory vaccination was not carried out against H9N2 AIV, but almost all chicken flocks were immunized with the H9 vaccine to reduce potential economic losses. According to the China National Veterinary Drug Basic Information Database, there have been approximately 50 biologic companies nationwide that have manufactured monovalent or multivalent H9N2 vaccines within the last 5 years. Among the certified H9 vaccine-related products, there were as many as 25 vaccine strains, and all of them were inactivated whole-virus vaccines (IWVs) (Table 1). Research and clinical data show that H9N2 vaccination can provide effective protection for immunized flocks by reducing clinical signs caused by virus infection. However, the frequent antigenic drift of H9N2 is one of the challenges of current vaccination against H9N2. IWV does not provide sufficient protection when there are differences in antigenicity between the H9N2 vaccine strain and the circulating strain [35,59]. The H9N2 vaccine needs to be optimized to fit the current co-epidemic status of multiple antigenic groups. Importantly, the inactivated H9N2 vaccine mainly induces humoral immunity, which makes it difficult to interrupt virus infection and shedding in the chicken upper respiratory tract. H9N2 AIV strains are capable of efficient chicken-to-chicken aerosol transmission, even among vaccinated chickens [60], increasing the difficulty of virus prevention and control. Therefore, ‘preventing shedding’ has become a new criterion and another challenge for the development of novel H9N2 vaccines. There is an urgent need to develop more effective vaccines to prevent and control H9N2, such as improving existing vaccines at the levels of cellular and mucosal immunity and developing universal vaccines to control the presence of multiple antigenic groups.

## 4. Vaccine Development against H9N2-Subtype Avian Influenza

### 4.1. Inactivated Whole-Virus Vaccines

As the most widely used type of influenza-virus vaccine in both humans and birds, IWV’s clinical effectiveness is unquestionable. IWV offers some advantages compared to other types of vaccines, including ease of production and its lack of ability to revert to a virulent state. Vaccine candidate strains determine the immunogenicity of the vaccine. The compatibility between HA and NA, the effect of HA deglycosylation and protective antigenic epitopes should be considered to screen better candidate strains (Table 2) [61]. 

Given that multiple H9N2 antigenic groups were prevalent in China, rapid preparation of an antigen-matched vaccine or effective prediction of antigen variation would be crucial for the control of H9N2. Antigenicity prediction models based on HA sequences show good potential [62]. Strains with broad-spectrum cross-protection may exist among the naturally prevalent strains, as reported for H7N9 subtypes of AIV [63]. An inactivated vaccine modified based on mosaic vaccine design ideas for the H9 gene was successfully prepared, but its broad-spectrum protection still needs to be further explored [64]. Epigraph, a graph-based vaccine-design algorithm, has been applied and demonstrated its broad protection against H3 subtypes [65,66]. In addition, a chimeric H9/H5N2 recombinant vaccine that expressed the whole HA1 region of H9N2 and the HA2 region of H5N8 viruses protected immunized chickens against lethal challenge by HPAI H5N8 viruses and significantly attenuated virus shedding after infection by both H9N2 and HPAI H5N8 viruses [67].

However, the H9N2 inactivated vaccine could not prevent immunized chickens from reinfection with H9N2 AIV and shedding virus. Many studies have tried to improve the immune protection of inactivated vaccines from multiple aspects. The virus used for producing inactivated vaccine is the main factor in inducing vaccine immunity. The seed virus used for vaccine preparation needs to be screened to better meet the immunogenicity of the current epidemic strain, which has cross-immune protection characteristics. For production purposes, vaccine candidate strains should replicate effectively in embryonated chicken eggs. At present, the construction of H9N2 vaccine candidate strains mostly uses PR8 (H1N1) as the backbone and recombines the HA and NA genes of epidemic viruses. This PR8 backbone has also been shown to improve titers in embryonated chicken eggs, a common propagation system for influenza viruses [68]. Alternatively, some scholars used the H9N2 virus to directly produce vaccine virus by screening the H9N2 virus, optimizing the virus sequence and increasing the virus load and its antigenicity [69,70]. 

The IWV is mainly prepared from the formaldehyde inactivation of virus-containing allantoic fluids from infected chicken embryos. Recently, several virus inactivation methods for producing influenza IWVs, including formaldehyde, treatment with beta-propiolactone (BPL) and the application of gamma radiation, have been analyzed for their immune protection effects. A study showed that antibody-mediated immune responses were increased in chickens that received BPL and gamma IWVs compared to formaldehyde IWV against H9N2 AIV [71]. Inactivated vaccines mainly induce humoral immunity. Adjuvants could improve the cellular immunity or mucosal immunity induced by IWV. CpG oligodeoxynucleotides assist the whole inactivated H9N2 influenza virus in crossing the intestinal epithelial barriers via transepithelial uptake of dendritic cell dendrites [72,73]. Similarly, bursopentine [74], bursin-like epitope peptide [75], poly I:C [76], bursal peptides [77], chitosan [78], *Bacillus subtilis* spores [79] and polyethyleneimine-coated PLGA nanoparticles [80] as adjuvants induce antigen-specific antibody and T-cell responses in poultry against H9N2 AIV. However, these adjuvants have not been used in the clinic so far.

### 4.2. Vector Vaccine

Advances in molecular biology have allowed a number of different vectored vaccines to be developed and licensed for use in the control of avian influenza (Table 2) [107]. Vector vaccines can induce additional cellular immunity and mucosal immunity through infection to provide good immunity. Recently, a variety of vectors have been used for H9 vaccine development, including fowlpox virus (FPV), fowl adenoviruses (FAdVs), Marek’s disease virus (MDV) and NDV. In addition to viral vectors, *Lactobacillus* [108,109,110,111] and *Eimeria acervuline* [112] are also used as vectors to prepare vaccines to prevent and control H9N2 avian influenza. However, overcoming maternal antibody interference is a major challenge.

Attenuated fowlpox virus (FPV) strains have been used as vaccines for decades to prevent wild-type virus infection. Recombinant FPVs have been developed and evaluated to prevent viral and mycoplasma infections in birds, and some have been licensed for their commercialization. Recombinant fowlpox virus (rFPV-HA) expressing the HA gene of H9N2 AIV-vaccinated groups could prevent virus shedding and replication in multiple organs in response to H9N2, and coexpression of IL18 enhanced the inhibition of viruses compared with the rFPV-HA-vaccinated group [81]. However, the efficacy of recombinant FPV-based vaccines can be affected by preexisting immunity [82].

Fowl adenoviruses (FAdVs), with a linear, 26–45 kb, double-stranded DNA molecule, can also be used as virus vectors. By inserting the nucleotide sequence encoding the VP2 protein of IBDV into rFAdV, rFAdV-VP2 was generated [83]. These authors evaluated the protection induced by rFAdV-VP2 in SPF chickens and found that rFAdV-VP2 vaccination induced good immune protection, suggesting its potential in the vector vaccine development of H9N2-subtype avian influenza virus. Currently, none of the recombinant FAdVs are being commercialized as vectored vaccines. Preexisting immunities hamper the application of FAdV vectors in vaccine development.

Marek’s disease virus (MDV) belongs to the family *Herpesviridae*, subfamily *Alphaherpesvirinae*, members of the *genus Mardivirus*, which are divided into three species, *gallid* herpesvirus 2, *gallid* herpesvirus 3 and *meleagrid* herpesvirus 1, formally named MDV serotype 1 (MDV-1), MDV serotype 2 (MDV-2) and MDV serotype 3 (MDV-3), respectively. Turkey herpesvirus (HVT), belonging to the meleagrid herpesvirus 1 family, has been extensively used as a vaccine against Marek’s disease for over 40 years. Attenuated MDV-1 strains and HVT have several characteristics that make them appropriate for the development of recombinant vector-based vaccines for poultry diseases. The attenuated CVI988/Rispens MDV-1 strain has been used to express the S1 glycoprotein of IBV [113] and the VP2 of IBDV [114]. Coding sequences for protective antigens of IBDV [115], avian leukosis virus [116] and AIV [117] were also inserted into the genome of the avirulent 814 MDV-1 strain. The efficacy of the recombinant MDV-1 strain 814 expressing AIV-H5 glycoprotein (rMDV-H5) and an HVT expressing the same antigen (rHVT-H5) was compared by challenge with the virulent MDV-1 (J-1) and AIV (HPAI H5N1 A/Goose/HLJ/QFY/03) strains [117]. The results showed that protection against AIV in chickens vaccinated with rHVT-H5 and rMDV-H5 was 66.7% and 80%, respectively. MDV-1 was also used to express HA of H9N2 AIVs (MDV-H9). Chickens vaccinated with MDV-H9 induced less than 50% protection [84]. The vector vaccine candidate HVT-H9 could induce robust humoral and cellular immunity in chickens. In a challenge study, no chicken shed H9N2 virus from the oropharynx and cloaca, and no H9N2 virus was found in the viscera in the vaccination groups when challenged with homologous virus, suggesting that HVT-H9 provides effective protection against H9N2 AIV in chickens [85]. 

Recombinant HVTs (rHVTs) encoding proteins of infectious laryngotracheitis virus (ILTV), IBDV, AIV and NDV have been commercialized as dual vaccines to control MDV and each of those pathogens. Vaccines based on HVT or MDV can be produced on mass by in ovo inoculation of the embryos or a subcutaneous route in one-day-old chickens. As these viruses are cell-associated, that is, display cell-to-cell transmission, they are not susceptible to maternal antibodies. In addition, MDV and HVT persistently infect their host, inducing lifelong immunity. Thus, HVT or MDV will be an important virus vector for H9N2 vaccine development. To date, HVT-H5 has been used for more than ten years, but there is no avian-influenza vector vaccine based on MDV-1 on the market. Similarly, duck-enteritis virus (DEV) was also used as a virus vector to construct a vector vaccine for the prevention and control of H9N2 virus in ducks, and DEV-H9 vaccination completely prevented the oropharyngeal shedding of H9N2 AIV [118]. This vector vaccine delivery was mainly through subcutaneous injection in the neck, which could not induce robust local mucosal immunity in the respiratory tract or provide better immune protection for AIVs.

NDV belongs to the family *Paramyxovidae* and genus *Avulavirus*. Its genome is a non-segmented, single-stranded, negative-sense RNA. In the poultry industry, naturally attenuated NDV strains are widely used to control NDV. It has been over 20 years since NDV was first used as a vector [119]. NDV-based viral vectors expressing the influenza NA and HA glycoproteins [86,120] have been obtained and evaluated as immunogens for chickens. Recombinant NDV expressing H9 HA protects SPF chickens against heterologous avian influenza H9N2 virus challenge [86,87,88,89]. However, interference from maternally derived antibodies greatly hinders the clinical application of these vaccines. APMV-2 belongs to the same genus as NDV, distantly related to NDV in the phylogenetic tree, based on the sequences of the fusion (F) and hemagglutinin-neuraminidase (HN) genes, and has low cross-reactivity with anti-NDV antisera. APMV-H9 conferred complete immune protection to prevent viral shedding in oropharyngeal and cloacal swabs from chickens challenged with H9N2 virus [90].

### 4.3. Live-Attenuated Vaccine

Live-attenuated AIV vaccines have been demonstrated to provide cross-protection against different influenza viruses (Table 2) [71]. In addition to inducing strong humoral immunity, it also elicited robust cellular immunity and mucosal immunity. Live-attenuated vaccines for humans are considered better than inactivated vaccines [121,122]. In poultry, attenuated cold-adapted live H9N2-subtype AIV vaccine strains have been shown to provide better protective efficacy [91,92,93]. Therefore, a live-attenuated vaccine has more advantages than an inactivated vaccine. However, there has been concern about the risks associated with reassortment events between vaccine strains and circulating wild-type viruses.

In recent years, many new technologies have been applied to develop novel live-attenuated vaccines. NS1 is the main viral protein responsible for counteracting the antiviral response, and it acts as an interferon (IFN) antagonist to suppress type-I IFN production while promoting viral replication [123]. Hence, recombinant influenza viruses with modified, truncated or absent NS1 are likely to be reasonable alternatives to generate live-attenuated influenza viruses, since they are attenuated in IFN-competent hosts [124]. Live-attenuated H9N2 vaccine produced by truncating the NS1 gene could also protect chickens against homologous and heterologous H9N2 AIV challenge [94,95]. In addition, reorganized PR8 viruses were constructed by splitting the overlapping open-reading frames of M1 and M2. Importantly, PR8 viruses that contained the M-split segment were highly attenuated in vivo, and they protected mice from a lethal homologous challenge with WT PR8 [125].

Codon-pair bias refers to the fact that some pairs of codons occur more frequently than others, and this frequency differs between species [126]. Using codon combinations that are less represented in the genetic code of the host could alter the expression of viral proteins and affect viral spread, thus generating live-attenuated influenza virus [127,128]. Synthetic attenuated-virus engineering was used to recode and synthesize the viral genome of PR8 [127] and A/California/7/2009 pandemic H1N1 (pH1N1) viruses [129] in a way that preserved the WT amino acid sequence but created a suboptimal arrangement of codon pairs. There were no significant differences in the growth kinetics or plaque phenotype of mutant deoptimized viruses compared to wild-type virus. In vivo analysis showed that the deoptimized viruses were remarkably attenuated in mice. Based on the premature termination codon (PTC), live-attenuated influenza viruses were also generated. PTC-harboring viruses exerted full infectivity but could not replicate in conventional cells. Vaccination with PTC viruses elicited robust humoral, mucosal, and T-cell-mediated immunity against antigenically distinct influenza viruses and even neutralized existing infecting strains [96]. In addition, single-cycle infectious IAVs can be generated by the mutation, deletion or substitution of viral components using molecular biology techniques. These viruses can be defective in viral genome synthesis, assembly or the release of viral particles and thus lack the ability to spread after initial infection [97]. A PR8 virus without the PB2 gene was shown to be safe in mice, and it was also immunogenic and protected mice from lethal challenge with PR8 [98], suggesting that this platform can be used to develop both monovalent and/or bivalent vaccines against influenza strains or different respiratory pathogens.

### 4.4. DNA and mRNA Vaccines

DNA and mRNA vaccines can use target sequences of clinical isolates as soon as they are available (Table 2). DNA or mRNA vaccines are also an effective vehicle for universal influenza vaccines. Based on highly conserved T-cell epitope studies, multiepitope DNA vaccines for the NP and M genes have been reported to be effective in protecting against multiple subtypes of influenza viruses in mice [130].

In combination with improved adjuvants to enhance humoral immune responses [131,132], DNA vaccines have been shown to be highly immunogenic and efficacious in poultry [99,133]. Chickens immunized with a DNA vaccine developed high levels of HI and NT antibodies and were completely protected from lethal H5 virus challenge. Importantly, the tri-clade DNA vaccine encoding HAs of clades 0, 2.3.2.1 and 7.2 elicited broadly neutralizing antibody responses against all H5 clades and subclades and protected mice against high-lethal-dose heterologous H5N1 challenge [133]. For chickens immunized with the H9N2 DNA vaccine, T lymphocytes were activated and proliferated, the numbers of CD3+, CD4+ and CD4+/CD8+ cells increased, and the chickens were completely protected against H9N2 AIV challenge [99]. Recently, DNA vaccines have been approved for the prevention of avian H5N1 in the USA and China.

mRNA-based vaccine platforms have been used to develop vaccines against infectious diseases, such as respiratory syncytial virus (RSV) [134], Zika virus [135], SARS-CoV-2 [136], Ebola virus [137] and HIV [138]. Importantly, mRNA-based vaccines have been licensed for commercial use against SARS-CoV-2. mRNA influenza vaccine constructs encoding the hemagglutinin of the H10N8 and H7N9 influenza strains formulated in a lipid nanoparticle delivery system induced HI and microneutralization titers when inoculated intramuscularly, although a significant T-cell response to vaccination was not found [100]. Although DNA or mRNA vaccines offer advantages, setbacks, including the inability to induce strong immunity and the fact that they are not currently applicable for mass vaccination, impede their use in the poultry industry.

### 4.5. Virus-like Particle Vaccine

Virus-like particles (VLPs) have a similar morphology to natural viruses but they lack any pathogenicity or infectivity (Table 2). With highly ordered epitope repeats, VLPs have excellent immunogenicity and can induce strong cellular and humoral immune responses [139]. VLP vaccines have mainly employed baculovirus-insect cell systems for production. Importantly, VLPs can avoid the biosafety threat caused by live viruses during the process of vaccine production. In addition, they can greatly reduce the costs due to the improvement of the production process. Experimentally, many different AIV VLP vaccines have been developed and shown to be highly immunogenic in chickens, including an H9N2 VLP [101], an H5N1 VLP [140], an H6N1 VLP [141] and an H7N9 VLP [142]. A single injection of the VLP vaccine induced high levels of HI antibodies and lowered the frequencies of virus isolation after wild-type virus challenge. VLPs are also widely used to develop universal vaccines. Triple-subtype VLPs that colocalized H5, H7 and H9 antigens derived from H5N1, H7N3 and H9N2 viruses were prepared and provided complete protection for H5N2 and H7N3 HPAIVs. The immune response was also detectable after challenge with H9N2 LPAIV [102,103]. The HA stalk and M2e are two potentially effective broad-spectrum immunogens against influenza, and displaying either on the surface using VLP technology can fulfill their protective potential as a universal vaccine against avian influenza viruses [143,144].

### 4.6. Recombinant Protein Vaccine 

Since recombinant protein vaccines are nonreplicating and lack any of the infectious components, they are considered a safer approach than vaccines derived from live viruses (Table 2). Baculovirus-insect cell systems or *E*. *coli* expression systems have been widely used to produce recombinant protein vaccines, which greatly reduces the production cost. However, a weak cellular immune response and no mucosal immune response prevent them from providing good protection. A recombinant protein vaccine can enhance the protective effect by mixing the main immune antigens with proteins that stimulate immune cells. The complete HA protein of the H5N2 virus was chemically conjugated to an anti-chicken Dec205 monoclonal antibody, and a single dose of this vaccine was shown to be sufficient to elicit a strong antibody response in chickens as early as fourteen days after initial immunization [104]. Furthermore, targeting a synthetic peptide antigen to the chicken CD40 receptor showed accelerated and enhanced antibody responses against the peptide antigen compared to untargeted peptide [145]. 

In addition, pattern recognition receptors targeting recombinant vaccines have also been investigated in chickens. An H7HA influenza subunit vaccine recombinantly fused to *Salmonella typhimurium* flagellin (H7HA-fliC) was generated. The immunization of chickens with H7HA-fliC showed robust antibody responses leading to a significant reduction in viral loads compared to the chickens receiving only H7HA [146]. Similarly, recombinant H9HA1-fliC enhances adherence to respiratory epithelial cells and promotes superior protective immune responses against H9N2 influenza virus in chickens [105]. CD83 is thought to play important roles during interactions between cells of the immune system and in B-cell function for antibody production in response to influenza A virus infection [106]. Recently, the H9N2 avian influenza virus hemagglutinin (HA) antigen was targeted by fusing it to single-chain fragment-variable (scFv) antibodies specific to the CD83 receptor expressed on chicken APCs. Following this, the vaccine-induced cellular and humoral immunity in chickens was compared to untargeted H9HA. Chickens vaccinated with CD83 scFv-targeted H9HA showed reduced mortality from an H9N2 challenge virus [106]. It can induce strong humoral immunity due to the high concentration of antigen. In addition, conserved proteins or conserved fragments of functional proteins have been used to develop universal vaccines. M2e is poorly immunogenic because of its small size, but its protective effect can be greatly enhanced by embedding it with antibodies that target specific immune cells, resulting in a universal vaccine [147,148]. To improve its immunogenicity while maintaining its original stability, chimeric HA and hyperglycosylated HA concepts have been applied to induce targeted HA-stalk immunity. This modified HA protein achieves a wide range of protective effects against at least one subtype [149,150,151].

## 5. Conclusions and Perspective

Currently, vaccination is still one of the principal strategies to control H9N2 avian influenza in China. However, a variety of antigenic H9N2 viruses are prevalent in China, and the vaccine development speed lags behind the speed of virus mutation, leading to a challenge for effective vaccination. Therefore, the development of a universal vaccine with broad-spectrum neutralizing activity is of great significance for the control of H9N2. Inactivated vaccine immunization can effectively reduce the clinical symptoms after H9N2 virus infection, greatly reducing economic losses. However, another challenge is that immunization with IWV cannot block H9N2-AIV reinfection or virus shedding. Live-attenuated vaccines can provide comprehensive immune protection, mainly because induced mucosal immunity plays an important role, indicating that vaccine-induced local mucosal immune responses, especially tissue resident T lymphocytes, play an important role in influenza virus immune protection in humans and mice. Many vaccines designed based on the mucosal immune response induce good mucosal immune effects and provide good immune protection in humans [152,153,154]. It is necessary to develop novel vaccines, especially those that can induce cellular immunity and local mucosal immunity, to control the prevalence of H9N2 AIV. In China, the traditional livestock-raising systems, including free-ranging and polyculture, continue to maintain their vital status. However, the main development direction of the Chinese poultry industry is moving toward intensive confinement and feeding. In addition to enhancing the mucosal immune response, improving the immune response to H9N2 in double or multiple combined vaccines also needs to be considered along with simplifying the immune procedure to reach an ideal goal of “one injection preventing multiple diseases”. To date, a large number of genetically engineered avian influenza vaccines have been designed in China. However, most of them have not been applied clinically. Developing multivalent live-vector vaccines or improving the protective effect of H9N2-multivalent inactivated vaccines will significantly contribute to the prevention and control of H9N2.

## Figures and Tables

**Table 1:** Commercial vaccines against H9 subtype avian influenza in China.

Vaccine Strain Abbreviation	Commercial Vaccine	Announcement No.	Announcement Date	Notes
F strain	AI (H9 subtype) IV (F/98 strain)	N/A	N/A	A/Chicken/Shanghai/F/98
	ND and AI (H9 subtype) combined IV (La Sota strain + F strain)	N/A	N/A	
HN03 strain	AI (H9 subtype) IV (HN03 strain)	2534	23 May 2017	N/A
HN106 strain	AI (H9 subtype) IV (HN106 strain)	2306	8 October 2015	A/chicken/Henan/01/2006
	ND and AI (H9 subtype) combined IV (La Sota strain + HN106 strain)	2390	15 April 2016	
	ND, IB and AI (H9 subtype) triple IV (La Sota strain + M41 strain + HN106 strain)	1489	26 November 2010	
	ND, IB, ED and AI (H9 subtype) quadruple IV (La Sota strain + M41 strain + HE02 strain + HN106 strain)	1883	7 January 2013	
LG1 strain	AI (H9 subtype) IV (LG1 strain)	N/A	N/A	A/Chicken/Shandong/LG1/2000
	ND and AI (H9 subtype) combined IV (La Sota strain + LG1 strain)	1322	11 January 2010	
	ND, IB, and AI (H9 subtype) triple IV (La Sota strain + M41 strain + LG1 strain)	1507	30 November 2010	
NJ01 strain	AI (H9 subtype) IV (NJ01 strain)	1938	6 May 2013	A/chicken/Nanjing/01/99
SD696 strain	AI (H9 subtype) IV (SD696 strain)	N/A	N/A	A/Chicken/Shandong/6/96
SS strain	AI (H9 subtype) IV (SS/94 strain)	N/A	N/A	A/Chicken/Guangdong/SS/94
	ND and AI (H9 subtype) combined IV ((La Sota strain + SS/94 strain)	N/A	N/A	
	ND, IB and AI (H9 subtype) triple IV (LaSota strain + M41 strain + SS/94 strain)	1530	19 January 2011	
	ND, IB, ED and AI (H9 subtype) quadruple IV (La Sota strain + M41 strain + K-11 strain + SS/94 strain)	2083	26 March 2014	
SY strain	AI IV (H9 subtype, SY strain)	N/A	N/A	A/chicken/Shaanxi/SY/97
	ND and AI (H9 subtype) combined IV (La Sota strain + SY strain)	1821	22 August 2012	
	ND, IB and AI (H9 subtype) triple IV (La Sota strain + M41 strain + SY strain)	1779	4 June 2012	
SZ strain	AI (H9 subtype) IV (SZ strain)	2270	24 June 2015	A/chicken/Shandong/SZ/2008
	ND and AI (H9 subtype) combined IV (La Sota strain + SZ strain)	2506	14 March 2017	
	ND, AI (H9 subtype) and IBD triple IV (La Sota strain + SZ strain + rVP2 protein)	2525	3 May 2017	
	ND, IB, AI (H9 subtype) and IBD quadruple IV (La Sota strain + M41 strain + SZ strain + rVP2 protein)	2400	5 May 2016	
HL strain	ND and AI (H9 subtype) double IV (La Sota strain + HL strain)	N/A	N/A	HL strain isolated from clinical cases in Luoyang, Henan, China in 2001
	ND, IB and AI (H9 subtype) triple IV (La Sota strain + M41 strain + HL strain)	N/A	N/A	
	ND, IB, ED and AI (H9 subtype) quadruple IV (La Sota strain + M41 strain + AV127 strain + HL strain)	N/A	N/A	
HP strain	ND and AI (H9 subtype) combined IV (La Sota strain + HP strain)	N/A	N/A	HP strain was isolated from diseased chicken flocks in Puyang, Henan, China in 1998
	ND, IB and AI (H9 subtype) triple IV (La Sota strain + M41 strain + HP strain)	1335	1 February 2010	
	ND, IB, ED and AI (H9 subtype) quadruple IV (La Sota strain + M41 strain + Z16 strain + HP strain)	N/A	N/A	
JD strain	ND and AI (H9 subtype) combined IV (La Sota strain + JD strain)	2577	31 August 2017	N/A
WD strain	ND and AI (H9 subtype) combined IV (La Sota strain + WD strain)	136	1 February 2019	WD strain was isolated from Wangdu, Hebei, China in 1998
	ND, IB and AI (H9 subtype) triple IV (La Sota strain + M41 strain + WD strain)	1489	26 November 2010	
	ND, IBD and AI (H9 subtype) triple IV (La Sota strain + BJQ902 strain + WD strain)	2557	2 August 2017	
	ND, IB, ED and AI (H9 subtype) quadruple IV (La Sota strain + M41 strain + HSH23 strain + WD strain)	2268	17 June 2015	
G strain	ND and AI (H9 subtype) combined IV (aSG10 strain + G strain)	164	16 April 2019	A/chicken/Hebei/G/2012
WJ57 strain	ND and AI (H9 subtype) combined IV (A-VII strain + WJ57 strain)	registered	15 October 2018	A/chicken/Jiangsu/WJ57/2012
D1 strain	DP and AI (H9 subtype) double IV (AV1221 strain + D1 strain)	2557	2 August 2017	N/A
HZ strain	ND, IB and AI (H9 subtype) triple IV (La Sota strain + M41 strain + HZ strain)	1556	13 April 2011	N/A
	ND, IB, ED and AI (H9 subtype) quadruple IV (La Sota strain + M41 strain + HS25 strain + HZ strain)	2106	28 May 2014	
JY strain	ND and AI (H9 subtype) combined IV (La Sota strain + JY strain)	1507	30 November 2010	A/chicken/Jiangsu/JY/99
L strain	ND, IB and AI (H9 subtype) triple IV (La Sota strain + M41 strain + L strain)	N/A	N/A	N/A
NJ02 strain	ND, IB and AI (H9 subtype) triple IV (La Sota strain + M41 strain + NJ02 strain)	1448	27 August 2010	A/chicken/Nanjing/02/2001
	ND, IB, ED and AI (H9 subtype) quadruple IV (La Sota strain + M41 strain + AV127 strain + NJ02 strain)	1548	4 March 2011	
Re9 strain (HuN33 strain)	ND, IB and AI (H9 subtype) triple IV (La Sota strain + M41 strain + Re9 strain)	2324	18 November 2015	A/chicken/Hunan/33/2008
	ND, AI (H9 subtype) and ED triple IV (La Sota strain + Re9 strain + Jing 911 strain)	164	16 April 2019	
YBF003 strain	ND, IB and AI (H9 subtype) triple IV (La Sota strain + M41 strain + YBF003 strain)	N/A	N/A	N/A
	ND, IB, ED and AI (H9 subtype) quadruple IV (La Sota strain + M41 strain + NE4 strain + YBF003 strain)	1908	1 March 2013	
	ND, AI (H9 subtype) and IBD triple IV (La Sota strain + YBF003 strain + VP2 protein)	1865	3 December 2012	
	ND, IB, AI (H9 subtype) and IBD quadruple IV (La Sota strain + M41 strain + YBF003 strain + SVP-2 protein)	1532	21 January 2011	
S2 strain	ND, IB, ED and AI (H9 subtype) quadruple IV (La Sota strain + M41 strain + AV-127 strain + S2 strain)	1532	21 January 2011	A/chicken/Shandong/S2/2005
YT strain	ND, AI and IC triple IV (La Sota strain + YT strain + DY2 strain)	582	24 July 2022	N/A
	ND, AI and IC triple IV (La Sota strain + YT strain + QD strain)	463	29 August 2021	
YBF13	ND, AI and IC triple IV (La Sota strain + YBF13 strain + YBAV-4 strain)	441	29 June 2021	N/A

Data source: China Institute of Veterinary Drug Control. N/A indicates that data are not available. Abbreviations: AI: avian influenza; ND: Newcastle disease; IB: infectious bronchitis; ED: egg-drop syndrome; DP: duck plague; IBD: infectious bursal disease; IC: infectious coryza; AA: avian adenovirus disease; IV: inactivated vaccine.

**Table 2:** Development and application of various types of vaccines.

Type of Vaccine	Development and Application	Notes	References
Inactivated whole-virus vaccines	Change vaccine strains	Enhanced vaccine compatibility	[61]
	Universal vaccine	Provides cross-protection	[63,64,65,66,67]
	Recombinant with PR8 virus	Improved production efficiency	[68]
	Gene modification	Modified HA sequence	[69,70]
	Inactivation method	Antibody-mediated immune responses were increased in chickens that received the BPL and gamma IWVs compared to the formaldehyde IWV	[71]
	Development of new adjuvants	CpG oligodeoxynucleotides, bursopentine, bursin-like epitope peptide, poly I:C, bursal peptides, chitosan, *Bacillus subtilis* spores, polyethylenimine-coated PLGA nanoparticles	[72,73,74,75,76,77,78,79,80]
Vector vaccine	Fowlpox virus	Affected by preexisting immunity	[81,82]
	Fowl adenoviruses	Application in IBD, not yet developed in influenza	[83]
	Marek’s disease virus	Could not induce robust local mucosal immunity in the respiratory tract	[84,85]
	Newcastle-disease virus	Interference from maternal antibodies greatly hinders clinical application	[86,87,88,89]
	APMV-2	Conferred complete immune protection	[90]
Live-attenuated vaccine	Attenuated cold-adapted live H9N2-subtype AIV vaccine	Provides better protection, but carries a biological risk	[91,92,93]
	Recombinant influenza virus with modified, truncated or absent NS1	Effectively reduced viral replication	[94,95]
	Codon-pair bias	Effectively reduced viral replication	[96,97,98]
DNA and mRNA vaccine	DNA vaccines	Conferred complete immune protection	[99]
	mRNA vaccine	Inability to induce strong immunity and unsuitable for mass vaccination	[100]
Virus-like particle vaccine	H9N2 VLP	Reduced biosecurity threats and costs	[101]
	Universal vaccination	Provides cross protection	[102,103]
Recombinant protein vaccine	conjugated to anti-chicken Dec205 monoclonal antibody	Strong immune protection 14 days after initial immunization	[104]
	H9 HA1-fliC	Promotes superior protective immune responses	[105]
	Chickens vaccinated with CD83 scFv targeted H9 HA	Effectively reduced viral replication	[106]

## Data Availability

Not applicable.
